# Anekomochi glutinous rice provides low postprandial glycemic response by enhanced insulin action via GLP-1 release and vagal afferents activation

**DOI:** 10.1186/s12576-024-00940-5

**Published:** 2024-09-27

**Authors:** Kento Ohbayashi, Yudai Sugiyama, Taichi Nohmi, Kazusa Nishimura, Tetsuya Nakazaki, Yo-Ichiro Sato, Takehiro Masumura, Yusaku Iwasaki

**Affiliations:** 1 Laboratory of Animal Science, Graduate School of Life and Environmental Sciences, Kyoto Prefectural University, 1-5 Hangi-Cho, Shimogamo, Sakyo-Ku, Kyoto, 606-8522 Japan; 2 Graduate School of Environmental, Life, Natural Science and Technology, Okayama University, 1-1-1 Tsushimanaka, Kita-Ku, Okayama, 700-0082 Japan; 3 Graduate School of Agriculture, Kyoto University, 4-2-1, Shiroyamadai, Kizugawa, Kyoto, 619-0218 Japan; 4 Office of Institutional Advancement and Communications, Kyoto University, Yoshida-Honmachi, Sakyo-Ku, Kyoto, 606-8501 Japan; 5 Research Center for Japanese Food Culture, Kyoto Prefectural University, 1-5 Hangi-Cho, Shimogamo, Sakyo-Ku, Kyoto, 606-8522 Japan; 6 Museum of Natural and Environmental History, Shizuoka, 5762 Oya, Suruga-Ku, Shizuoka, 422-8017 Japan; 7 Laboratory of Genetic Engineering, Graduate School of Life and Environmental Sciences, Kyoto Prefectural University, 1-5 Hangi-Cho, Shimogamo, Sakyo-Ku, Kyoto, 606-8522 Japan

**Keywords:** Glutinous rice, Postprandial glycemic response, GLP-1, Insulin, Vagal afferent nerves

## Abstract

Glutinous rice (*mochi* rice), compared to non-glutinous rice (*uruchi* rice), exhibits a wide range of glycemic index (GI) values, from low to high. However, the underlying mechanisms behind the variation in GI values remain poorly understood. In this study, we aimed to identify rice cultivars with a low postprandial glycemic response and investigate the mechanisms, focusing on insulin and incretin hormones. We examined seven glutinous rice cultivars and three non-glutinous rice cultivars. We discovered that Anekomochi, a glutinous rice cultivar, has the lowest postprandial glycemic response. Anekomochi significantly enhanced glucagon-like peptide-1 (GLP-1) secretion while suppressing insulin secretion. These effects were completely blunted by inhibiting GLP-1 receptor signaling and denervating the common hepatic branch of vagal afferent nerves that are crucial for sensing intestinal GLP-1. Our findings demonstrate that Anekomochi markedly enhances insulin action via GLP-1 release and vagal afferent neural pathways, thereby leading to a lower postprandial glycemic response.

**Supplementary Information:**

The online version contains supplementary material available at 10.1186/s12576-024-00940-5.

## Background

Postprandial hyperglycemia is associated with the development of type 2 diabetes by inducing insulin resistance and dysfunction of pancreatic β-cells [1, 2]. The glycemic index (GI) is a numerical indicator that quantifies the blood glucose-raising potential of a food after consuming a fixed amount of carbohydrates [3]. Numerous epidemiologic studies have shown that a high-GI diet correlates with an increased risk of developing type 2 diabetes [4–6]. On the other hand, a low-GI diet has been shown to be effective in the prevention and treatment of diabetes and obesity [7–11]. According to dietary guidelines for Japan and other countries, it is recommended that 45–65% of daily energy intake should come from carbohydrates [12–15]. Therefore, scientifically understanding methods of carbohydrate intake that do not cause a rapid increase in postprandial blood glucose levels is important for the prevention and treatment of diabetes.

White rice is a major grain and staple food, essential to the food culture in Asian countries, especially Japan. However, white rice is known for its high GI [16, 17]. Some reports indicate that high rice intake increases the risk of type 2 diabetes [18, 19]. Therefore, it is important to establish a method of consuming white rice that moderates the rise in blood glucose. Rice can be classified into two types based on starch structure: non-glutinous rice (*uruchi* rice, regular rice) and glutinous rice (*mochi* rice, waxy rice). Non-glutinous rice contains about 20% amylose and 80% amylopectin, while glutinous rice is almost 100% amylopectin. Amylopectin is rapidly broken down by digestive enzymes and is thought to significantly impact postprandial blood glucose levels. In fact, it has been reported that glutinous rice has a greater postprandial glycemic response than non-glutinous rice [16]. However, a systematic review published in 2021 shows that non-glutinous rice consistently has a high GI around 80, while glutinous rice exhibits a wide range of GI values between 48 and 94 [17]. These controversial effects of glutinous rice on blood glucose levels have not been conclusively determined.

The intestinal hormone glucagon-like peptide-1 (GLP-1) plays a crucial role in suppressing postprandial glucose elevation [20, 21]. GLP-1 is released from intestinal enteroendocrine L cells in response to macronutrients intake and enhances glucose-induced insulin secretion by directly acting on GLP-1 receptors in pancreatic β-cells [21, 22]. Endogenous intestinal GLP-1 is unstable due to degradation by dipeptidyl peptidase-4 (DPP-IV). Recent studies have shown that endogenous GLP-1 promotes not only insulin release but also insulin action through brain–systemic crosstalk initiated by the activation of vagal afferent nerves expressing GLP-1 receptors [23–26]. However, it remains unclear whether GLP-1 release or vagal afferents contribute to the differences in postprandial glycemic responses to rice.

In the present study, we aimed to identify rice cultivars with low postprandial glycemic responses among three non-glutinous cultivars widely consumed in Japan and seven glutinous rice cultivars selected to cover genetic variation, and to investigate whether these effects are mediated by GLP-1 and vagal afferent neural pathways. We quantified the starch content in all polished rice cultivars, administered a single oral dose of 2 g/kg total starch rice solution to mice, and measured changes in blood glucose levels over time. All three non-glutinous rice cultivars showed a high postprandial glycemic response, whereas the seven glutinous rice cultivars exhibited a range of responses from low to high. Anekomochi was identified as the glutinous rice cultivar with the lowest postprandial glycemic response. Peroral administration of Anekomochi significantly increased plasma GLP-1 levels while suppressing insulin levels. Furthermore, the low postprandial glycemic response of Anekomochi was completely blunted by pharmacological or genetic GLP-1 receptor blockade or denervation of the common hepatic branch of vagal afferent nerves. These results indicate that Anekomochi enhances insulin action by promoting GLP-1 secretion and activating vagal afferent neural pathways via the secreted GLP-1, resulting in suppressed postprandial blood glucose elevation.

## Methods

### Rice samples

Ten rice samples used in this study are listed in Table 1. We selected three non-glutinous rice cultivars (*uruchi* rice, regular rice), which are well-known and widely consumed in Japan. Hinohikari is primarily cultivated in western Japan. Kirara397 and Nanatsuboshi are primarily cultivated in Hokkaido. Seven glutinous rice cultivars (*mochi* rice, waxy rice) were selected to cover genetic variation that has been established in diverse natural and human environments. Unhulled rice was threshed to obtain brown rice using a rice huller (TR-250, Kett Electric Lab, Tokyo, Japan), and this brown rice was polished about 10% using a rice polisher (TP-3000, Kett Electric Lab, Tokyo, Japan). This polished rice was crushed using a blender and then further ground in a mortar and pestle to produce rice flour.

**Table 1:** Information on rice samples, their starch content, and dosage for the mice

Cultivar name	Abbreviation	Characteristics of glutinous	Starch content (%)	Dose (g/kg)
*Oryza sativa* L. cv Hinohikari	Hinohikari	Non-glutinous rice	77.15	2.59
*Oryza sativa* L. cv Kirara397	Kirara397	Non-glutinous rice	71.74	2.79
*Oryza sativa* L. cv Nanatsuboshi	Nanatsuboshi	Non-glutinous rice	75.18	2.66
*Oryza sativa* L. cv Shimizumochi	Shimizumochi	Glutinous rice	76.72	2.61
*Oryza sativa* L. cv Habutaemochi	Habutaemochi	Glutinous rice	71.90	2.78
*Oryza sativa* L. cv Khao ha noi	Khao ha noi	Glutinous rice	80.09	2.50
*Oryza sativa* L. cv Anekomochi	Anekomochi	Glutinous rice	73.72	2.71
*Oryza sativa* L. cv Akamochi	Akamochi	Glutinous rice	68.85	2.90
*Oryza sativa* L. cv Nioimochi	Nioimochi	Glutinous rice	69.64	2.87
*Oryza sativa* L. cv Hong Xie Nuo	Hong Xie Nuo	Glutinous rice	75.51	2.65

Three cultivars of non-glutinous rice (*uruchi* rice, regular rice) and seven cultivars of glutinous rice (*mochi* rice, waxy rice) were used in this study. The starch content of white rice obtained by 10% polishing of brown rice was quantified using the F-kit starch (R-Biopharm AG). Dosages were determined to standardize starch content to 2 g/kg for all groups

Total starch content in the rice samples was analyzed by standard chemical degradation methods using the F-kit starch (#207748, R-Biopharm AG, Darmstadt, Germany) (Table 1). The concentration of rice solution was determined based on the starch content in each rice cultivar, and the dosage of starch administered to the mice was 2 g/20 ml/kg (Table 1). On the day of the experiment, water was added to the rice flour to prepare rice solution.

### Animals

Male C57BL/6J mice were purchased from the Jackson Laboratory Japan, Inc. (Yokohama, Japan) and housed under controlled temperature (22.5 °C ± 2 °C), humidity (55% ± 10%), and light (light phase; 7:30–19:30). The purchased mice were acclimated to the new environment for at least one week before the experiments. *Glp1r*^−/−^ (*Glp1r* KO) mice on the C57BL/6J background generated as described previously [27] were kindly provided by Dr. Daniel J Drucker (Lunenfeld Tanenbaum Research Institute, Mt. Sinai Hospital, Tronto, Canada). Standard chow (CE-2, CLEA Japan, Tokyo, Japan) and water ad libitum were available to the mice. All experiments were performed on male mice between 9 and 19 weeks of age. The animal experiments were carried out after receiving approval from the Institutional Animal Experiment Committee of the Kyoto Prefectural University and in accordance with the Institutional Regulations for Animal Experiments (approval number: KPU040907-4C).

### Sequential blood glucose measurement following peroral administration of rice solution

Mice were fasted overnight (18:00 to next 10:00). D-Glucose solution (2 g/20 ml/kg) or rice solutions (2 g/20 ml/kg, as total starch content) were administered perorally (po) into the stomach using a stainless steel feeding needle at 10:00. Subsequently, blood samples were collected from the tail vein at 15–120 min using heparinized capillary glass tubes. Glucose levels in the blood samples were determined using GlucoCard Plus Care (Arkray, Kyoto, Japan). Plasma insulin was measured by insulin ELISA kit (MS303, Morinaga, Yokohama, Japan). To examine the postprandial glycemic response of the 10 rice samples, glucose administration tests were performed in all experiments and the results were used for standardization. In experiments using a GLP-1 receptor antagonist, exendin (9–39) (600 nmol/5 ml/kg, Abcam, Cambridge, UK) or saline was intraperitoneally (ip) injected at 15 min before the po administration of rice solutions. Homeostatic model assessment of insulin resistance (HOMA-IR), an indicator of insulin action, was calculated as follows [26, 28]; insulin (ng/ml) × 26 × blood glucose (mg/dl)/405.

### Measurement of glucose, insulin, GLP-1, and GIP in poral vein plasma

Mice were fasted overnight (18:00 to next 10:00). Rice solutions (2 g/20 ml/kg, total starch) were po administered at 10:00. Then, the blood samples were collected from the portal vein under isoflurane anesthesia at 15 min after injection. The sampling syringe contained heparin (final concentration; 50 IU/ml), aprotinin (final concentration; 500 KIU/ml), and DPP-IV inhibitor vildagliptin (final concentration; 10 μM, for stable measurements). Plasma was collected after centrifugation (4000 rpm, 10 min at 4 °C) and stored at − 80 °C until assay. Plasma glucose, insulin, total GLP-1, and total GIP were measured using Glucose CII Test Wako (439-90901; Fujifilm Wako Pure Chemical Corporation, Osaka, Japan), Insulin ELISA kit (MS303, Morinaga), GLP-1 total ELISA kit (EZGLP1T-36K; Millipore, MA, USA), and GIP total ELISA kit (EZRMGIP-55K; Millipore), respectively.

### Surgical and chemical denervation of the common hepatic branch of the vagus nerve

Vagotomy of the common hepatic branch was performed as described [24, 29]. Briefly, a midline incision was made to obtain a wide exposure of the upper abdominal organs and the common hepatic branch of the vagus nerve in mice anesthetized with tribromoethanol (200 mg/kg, ip). Since the common hepatic vagal branch forms a neurovascular bundle, this branch was selectively ligated with silk sutures and cut using microscissors. Operated mice were recovered under standard chow diet for 1 week before experiments.

The chemical deafferentation of the common hepatic branch of the vagus nerve was performed as described [30, 31]. The common hepatic branch of the vagus nerve was exposed as described above under anesthesia. The common hepatic branch of the vagus was freed from the surrounding tissue, wrapped with parafilm to isolate the treatment area, and exposed to a cotton ball soaked in 5% wt/vol capsaicin solution (50 mg/ml, composed of 10% Tween80, 90% olive oil) for 30 min. These mice were recovered under standard chow diet for 1 week before experiments.

The function of the common hepatic branch of the vagus nerve affects lipid metabolism during fasting and the weight of epididymal white adipose tissue [32]. To verify the appropriateness of these techniques, we measured the weight of epididymal white adipose tissue during fasting and confirmed impairment of the common hepatic branch function (data not shown).

### Statistical analysis

All data were shown are means ± SEM. Statistical analysis was performed by two-tailed unpaired *t*-test or by one-way or two-way ANOVA. When ANOVA indicated a significant difference among groups, these groups were compared by Dunnett’s, Tukey’s or Bonferroni’s post hoc test. All statistical analyses were performed using Prism 10 (GraphPad Software, CA, USA). *p* < 0.05 was considered significant.

## Results

### Evaluation of postprandial glycemic response of seven glutinous rice cultivars and three non-glutinous rice cultivars: low postprandial glycemic response of the glutinous rice cultivar Anekomochi

**Fig. 1:**
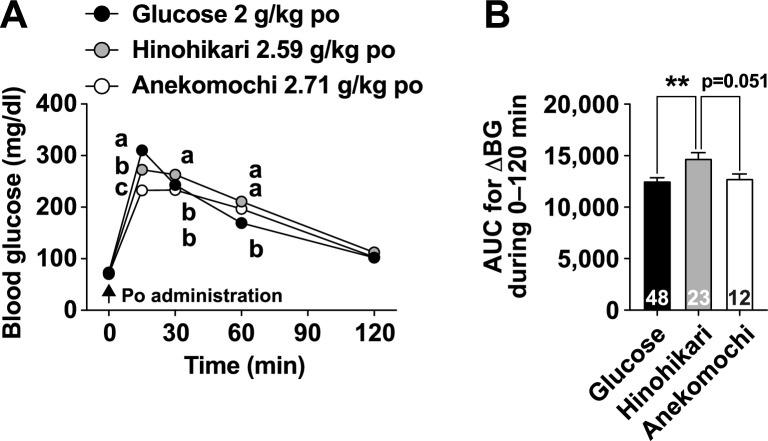
Changes in blood glucose levels after administration of aqueous solutions of glucose, non-glutinous rice (Hinohikari), and glutinous rice (Anekomochi) in mice. Blood glucose levels (**A**) and their area under the curve for the increase in blood glucose (AUC for ΔBG) during 0–120 min (**B**) after peroral (po) administration of glucose (2 g/kg, filled circles, n = 48), Hinohikari (2.59 g/kg, gray circles, n = 23), or Anekomochi (2.71 g/kg, open circles, n = 12) into the stomach of overnight-fasted C57BL/6 J mice using a stainless steel feeding needle. The starch content was standardized to 2 g/kg in all groups. The numbers inside the bars (**B**) indicate the sample size. Glucose group was included in all experiments conducted on different days as a positive control. The present data were selected from the results in Supplementary Fig. 1. Different alphabet letters within the same time point indicate *p* < 0.05 by two-way ANOVA followed by Tukey’s test in **A**, and **p < 0.01 by one-way ANOVA followed by Tukey’s test in **B**

First, the starch content in polished rice samples was quantified to determine a consistent amount of starch in the rice solutions administered to the mice. The results showed that the ten rice cultivars contained approximately 70–80% starch (Table 1). Next, to examine how each rice cultivar affects blood glucose levels, we perorally administered rice solutions standardized to a starch content of 2 g/kg or glucose at 2 g/kg to mice fasted overnight, and sequentially measured blood glucose levels up to 120 min after administration (Fig. 1 and Supplementary Fig. 1). Peroral (po) administration of glucose caused a transient increase in blood glucose levels, peaking at 15 min and returning to baseline at 120 min (Fig. 1A). Po administration of Hinohikari, a non-glutinous rice (*uruchi* rice), significantly suppressed the rapid rise in blood glucose levels 15 min after administration compared to the glucose group. However, blood glucose levels at 30 and 60 min after administration of Hinohikari were significantly higher than those of glucose (Fig. 1A), resulting in a higher area under the curve (AUC) for blood glucose during 0–120 min compared to the glucose group (Fig. 1B). In Anekomochi, a glutinous rice (*mochi* rice), blood glucose elevation at 15 and 30 min after administration was significantly lower than that in the Hinohikari group, and its AUC also tended to decrease compared to the Hinohikari group (Fig. 1A, B).

To evaluate the postprandial glycemic response of seven glutinous rice cultivars and three non-glutinous rice cultivars, we compared the glycemic response to each rice sample with the response to glucose administration (Supplementary Fig. 1). The glycemic responses, measured and calculated as the AUC for blood glucose increases during 0–120 min, for all three non-glutinous rice cultivars were significantly higher than the responses to glucose at 2 g/kg (Supplementary Fig  1A–C). On the other hand, the glycemic responses of the seven glutinous rice cultivars varied: Akamochi showed a significantly higher blood glucose response than glucose, Anekomochi tended to show a lower response, and others showed comparable levels (Supplementary Fig. 1D–J).

**Fig. 2:**
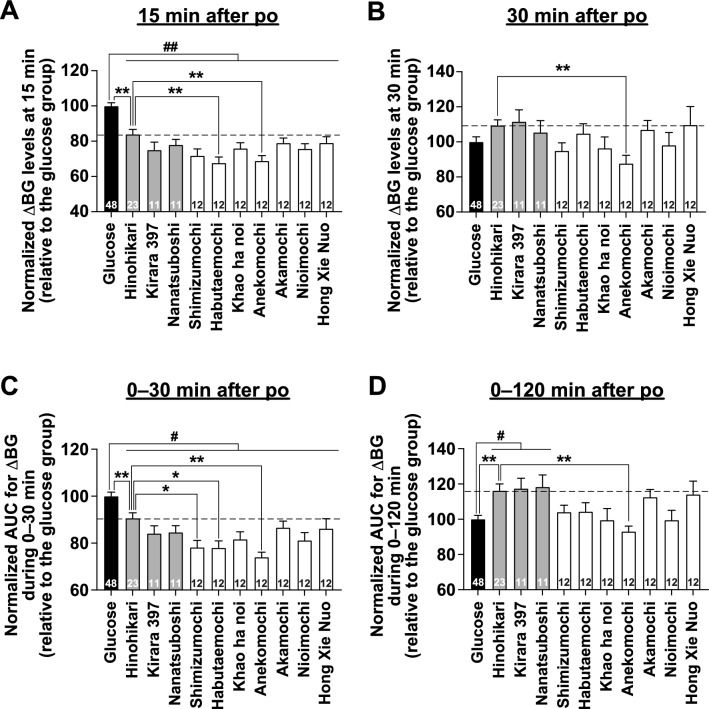
Comparison of blood glucose increase after administration of three non-glutinous rice cultivars, seven glutinous rice cultivars, and glucose in mice. Normalized change in blood glucose (ΔBG) levels at 15 min (**A**) and 30 min (**B**), and the AUC for the increase in blood glucose (AUC for ΔBG) during 0–30 min (**C**) and 0–120 min (**D**) after po administration of glucose (2 g/kg, filled bars, n = 48), three non-glutinous rice cultivars (2.59–2.66 g/kg, gray bars, n = 11–23), or seven glutinous rice cultivars (2.50–2.90 g/kg, open bars, n = 12) in C57BL/6J mice fasted overnight. The starch content was standardized to 2 g/kg in all groups. Glucose group was included in all experiments conducted on different days, and the ΔBG and AUCs of all rice-administered groups were normalized based on the results of the glucose group from experiments conducted on the same day. These data were obtained from the data in Supplementary Fig. 1. The numbers inside the bars indicate the sample size. **p < 0.01, *p < 0.05 by one-way ANOVA followed by Dunnett’s test (vs. Hinohikari group), and *##p* < 0.01*, #p* < 0.05 by one-way ANOVA followed by Dunnett’s test (vs. glucose group)

In Fig. 2, we normalized the blood glucose levels and the AUC for the increase in blood glucose following the po administration of glucose at 2 g/kg to compare the glycemic responses among the ten rice cultivars. At 15 min after administration, the relative blood glucose increase for all ten rice samples was significantly lower than that of glucose (Fig. 2A), however, the difference was no longer significant at 30 min (Fig. 2B). Based on these results, the relative AUCs during the 0–30 min period after administration for all ten rice samples were significantly lower than that for glucose (Fig. 2C). One notable point in these results is that Habutaemochi and Anekomochi, both glutinous rice cultivars, significantly lowered postprandial glycemic responses at 15 min compared to the non-glutinous rice Hinohikari (Fig. 2A). Furthermore, relative blood glucose levels at 30 min after administration of Anekomochi were also significantly lower than those of Hinohikari (Fig. 2B). Additionally, the relative AUCs during the 0–30 min and 0–120 min period after administration for Anekomochi were significantly lower than those for Hinohikari (Fig. 2C, D). These results indicate that Anekomochi has a low postprandial glycemic response potential compared to non-glutinous rice Hinohikari.

When assessed by the relative AUC during 0–120 min after administration, all three non-glutinous cultivars had similarly high values (Fig. 2D). On the other hand, the seven glutinous cultivars ranged from low to high (Fig. 2D). Among the glutinous rice cultivars, Anekomochi had the lowest glycemic response (Fig. 2D).

### Anekomochi promotes GLP-1 secretion while suppressing insulin secretion more potently than Hinohikari

**Fig. 3:**
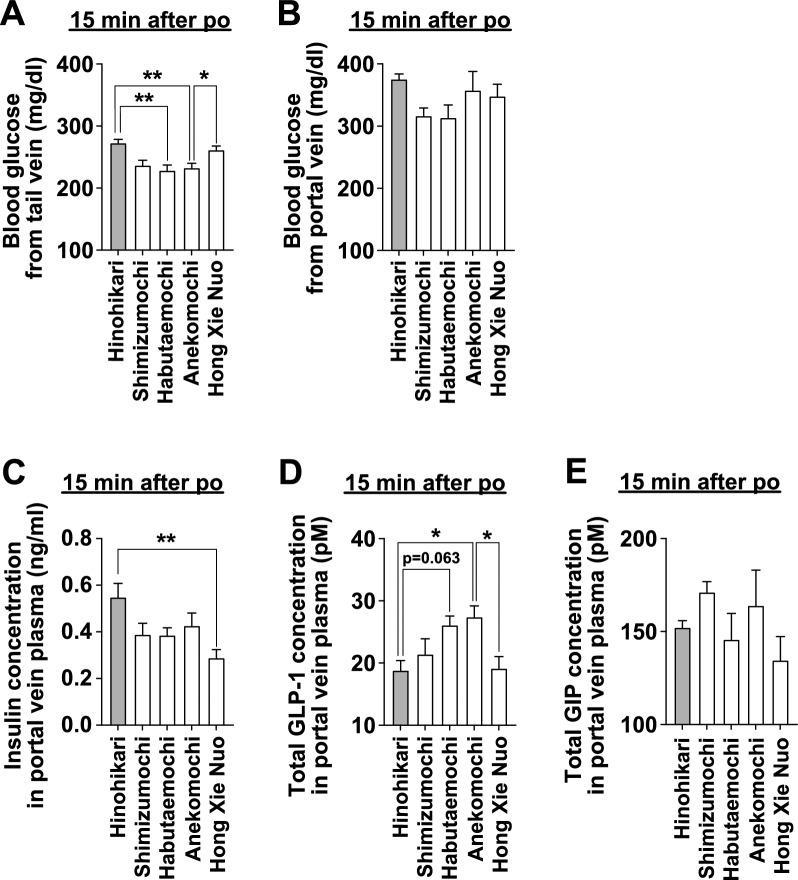
Anekomochi potentiates the increase in plasma GLP-1 levels more than Hinohikari. Fifteen min after po administration of the rice solution, blood was collected from the tail vein (**A**, conscious) or portal vein (**B–E**, anesthetized) in independent experiments. Hinohikari (2.59 g/kg), Shimizumochi (2.61 g/kg), Habutaemochi (2.78 g/kg), Anekomochi (2.71 g/kg), or Hong Xie Nuo (2.65 g/kg) were administered to C57BL/6 J mice fasted overnight. The starch content was standardized to 2 g/kg in all groups. Blood glucose (**A**, **B**), plasma insulin (**C**), total GLP-1 (**D**), and total GIP (**E**) concentrations were measured. n = 12–23 in **A** and n = 7–8 in **B**–**E**. **p < 0.01, *p < 0.05 by one-way ANOVA followed by Tukey’s test

We next examined the mechanisms by which some glutinous rice cultivars exhibit a low postprandial glycemic response. Blood samples were collected from the portal vein at 15 min after administration of each rice sample, and the concentrations of blood glucose and glucose metabolism-related hormones were measured (Fig. 3). In this experiment, we selected Hinohikari as the non-glutinous rice cultivar and four glutinous rice cultivars: those with a low glycemic response (Shimizumochi, Habutaemochi, and Anekomochi, Fig. 2) and one with a high glycemic response (Hong Xie Nuo, Fig. 2).

Blood glucose levels from the portal vein, which are parameters of the luminal digestion and absorption of rice starch, were comparable among the five groups (Fig. 3B). However, blood glucose levels in the tail vein, influenced by whole-body organ metabolism, were high in the Hinohikari and Hong Xie Nuo groups, and low in the Shimizumochi, Habutaemochi, and Anekomochi groups (Figs. 2A, 3A). These results suggest that the three glutinous rice cultivars with low glycemic response enhance glucose metabolism, but not due to a slower rate of starch absorption.

Portal vein insulin concentrations at 15 min after administration were high in the Hinohikari group, low in the Hong Xie Nuo group, and intermediate in the remaining three groups (Fig. 3C). Total GLP-1 concentrations in the portal vein were low in Hinohikari and Hong Xie Nuo groups, which show a high glycemic response, and high in the Shimizumochi, Habutaemochi, and Anekomochi groups, which show a low glycemic response (Figs. 2D vs. 3D). Notably, the total GLP-1 levels in the Anekomochi group were significantly higher than those in the Hinohikari and Hong Xie Nuo groups (Fig. 3D). Total GIP concentration did not differ between these groups (Fig. 3E). These data indicate that low glycemic response glutinous rice cultivars, especially Anekomochi and Habutaemochi, potently promote GLP-1 secretion without affecting insulin secretion.

### Anekomochi exhibits a low glycemic response by enhancing insulin action through GLP-1 receptor signaling

**Fig. 4:**
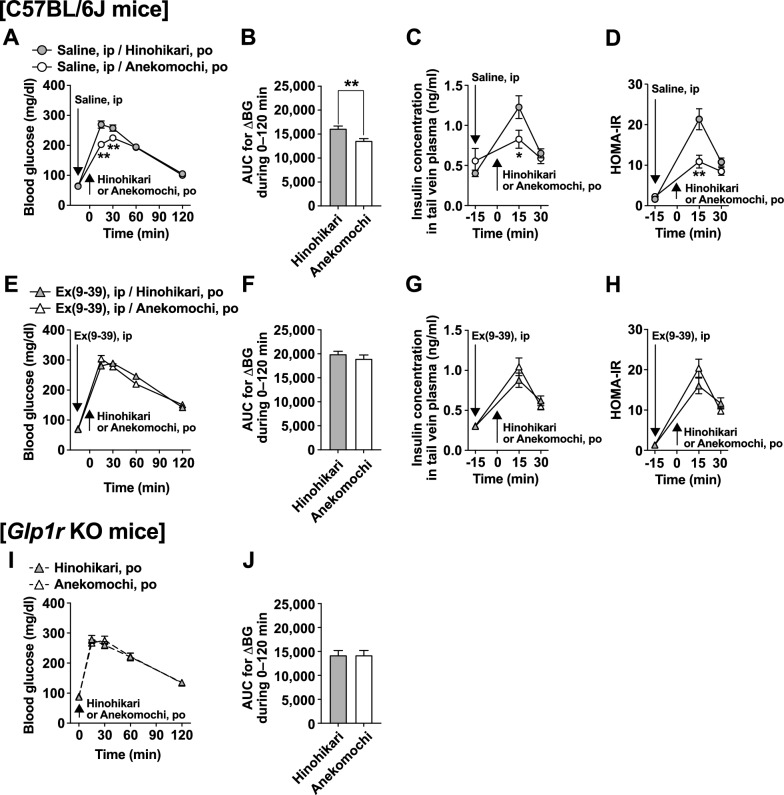
The low postprandial glycemic response of Anekomochi is due to GLP-1 receptor signaling. Saline (5 ml/kg, **A–D**) or GLP-1 receptor antagonist exendin(9-39) amide (Ex(9–39), 600 nmol/5 ml/kg, **E–H**) was ip injected 15 min prior to po administration of Hinohikari (2.59 g/kg) or Anekomochi (2.71 g/kg) in C57BL/6 J mice fasted overnight. Blood glucose (**A**, **E**) and plasma insulin (**C**, **G**) were measured, and AUCs for the increase in blood glucose (AUC for ΔBG) during 0–120 min (**B**, **F**) and HOMA-IR, an indicator of insulin action (**D**, **H**) were calculated. n = 9. Changes in blood glucose (**I**) and their AUC (**J**) after administration of Hinohikari or Anekomochi in *Glp1r* KO mice were measured. n = 11. **p < 0.01, *p < 0.05 by two-way ANOVA followed by Bonferroni’s test in **A**,** C, D**. **p < 0.01 by unpaired *t*-test in **B**

We examined whether the low postprandial glycemic effect of Anekomochi is mediated by GLP-1 receptor (GLP-1R) signaling. In the control experiment, saline (5 ml/kg) was intraperitoneally (ip) injected at -15 min, followed by the po administration of Anekomochi or Hinohikari at 0 min (Fig. 4A–D). Anekomochi significantly attenuated the rise in blood glucose levels (Fig. 4A) and decreased the AUC for blood glucose increase during 0–120 min (Fig. 4B) compared to the Hinohikari group. The rise in plasma insulin levels at 15 min after po administration was markedly lower in the Anekomochi group than the Hinohikari group (Fig. 4C), supporting the results in Fig. 3C. Additionally, HOMA-IR values, which are an indicator of whole-body insulin action, were also notably reduced in the Anekomochi group at 15 min after po administration (Fig. 4D). These results suggest that Anekomochi enhances insulin action, rather than insulin secretion, resulting in an improved postprandial rise in blood glucose effect. The Anekomochi-induced suppression of blood glucose elevation and plasma insulin elevation was completely abolished by pretreatment with the GLP-1R antagonist exendin(9–39) amide (Ex(9–39), 600 nmol/5 ml/kg, ip) (Fig. 4E–H) and in global GLP-1R knockout mice (*Glp1r* KO mice) (Fig. 4I, J). Habutaemochi, which tends to promotes GLP-1 secretion (Fig. 3D), exhibited a low glycemic response in control C57BL/6J mice, but not in in *Glp1r* KO mice (Supplementary Fig. 2). These results demonstrate that glutinous rice cultivars Anekomochi and Habutaemochi enhance insulin action via the GLP-1R signaling, thereby exhibiting a low postprandial glycemic response.

### The common hepatic branch of the vagal afferent nerves is essential for low postprandial glycemic response by Anekomochi

**Fig. 5:**
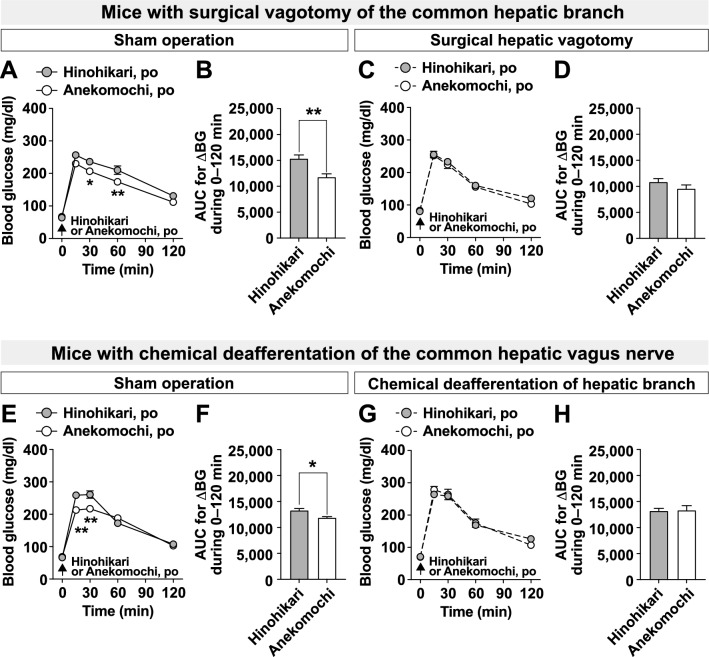
The vagal afferents of the common hepatic branch are essential for the low postprandial glycemic response of Anekomochi. Hinohikari (2.59 g/kg) or Anekomochi (2.71 g/kg) was po administered to sham-operated mice (**A**,** B**, n = 6; **E**,** F**, n = 5), surgically hepatic vagotomized mice (**C**, **D**, n = 6), or mice with chemical deafferentation of the common hepatic vagus nerve (**G**,** H**, n = 6), which were fasted overnight. Blood glucose was measured, and the AUCs for the increase in blood glucose (AUC for ΔBG) during 0–120 min were calculated. **p < 0.01, *p < 0.05 by two-way ANOVA followed by Bonferroni’s test in **A**,** E**. **p < 0.01, *p < 0.05 by unpaired *t*-test in **B**,** F**

Previous studies have reported that endogenous GLP-1 acts on vagal afferent nerves expressing GLP-1R to regulate glucose metabolism [23, 24, 26]. Additionally, the common hepatic branch of vagal afferents is crucial for sensing gastrointestinal hormones such as GLP-1 [24, 33]. Therefore, we finally explored whether the common hepatic branch of vagal afferents is involved in the low postprandial glycemic effect of Anekomochi. Oral administration of Anekomochi attenuated the increase in blood glucose levels and its AUC compared to Hinohikari in sham-operated mice (Fig. 5A, B, E, F). The low postprandial glycemic effect of Anekomochi was completely blunted in surgically hepatic vagotomized mice, which have impaired both afferent and efferent nerves (Fig. 5C, D), and in mice with chemical deafferentation of the common hepatic vagus nerve using capsaicin (Fig. 5G, H). Collectively, our results indicate that Anekomochi potently induces GLP-1 secretion to activate the common hepatic branch of vagal afferent nerves and enhance insulin sensitivity, resulting in a lower postprandial glycemic response.

## Discussion

In the present study, we found that seven glutinous rice cultivars exhibited a wide range of postprandial blood glucose responses, from low to high, whereas all three non-glutinous rice cultivars consistently showed a high glycemic response. Among the ten rice samples examined, Anekomochi was identified as the glutinous rice cultivar with the lowest postprandial glycemic response. Anekomochi induced the highest increase in blood GLP-1 levels and suppressed postprandial glycemic response by enhancing insulin action rather than insulin secretion. The mechanism behind Anekomochi’s low postprandial glycemic response involved GLP-1 receptor signaling and the common hepatic branch of vagal afferent nerves expressing GLP-1 receptor. Our findings reveal that glutinous rice cultivars such as Anekomochi have the ability to improve glucose tolerance, possibly due to their ability to stimulate GLP-1 secretion.

The main component of both polished glutinous rice and non-glutinous rice is starch, with their primary difference being the starch structure. The starch in glutinous rice is composed of 100% amylopectin, which varies in its branched structure across different cultivars [34–36]. Amylopectin, with its highly branched structure compared to amylose, is easily digested and reportedly causes a high glycemic response [16]. In contrast, several human studies have reported that glutinous rice exhibits a wide range of GI values, with some showing low GI values [17]. Our present investigation using a mouse model supports previous reports: po administration of the seven glutinous rice cultivars showed various postprandial glycemic responses. Furthermore, the beneficial effects of glutinous rice with low GI were related to GLP-1 and its regulation of glucose metabolism, not due to differences in the rates of starch digestion or absorption. The amylopectin-derived starch branching structure in glutinous rice varies across cultivars [34–36], which might result in diverse structures of the oligosaccharides produced during luminal digestion. A recent review indicates that various oligosaccharides have beneficial physiological functions such as lowering blood glucose [37]. Therefore, the degradation products of Anekomochi starch may include oligosaccharides with active structures that promote GLP-1 secretion, potentially resulting in a low postprandial glycemic response. This hypothesis needs to be further explored in future studies.

This study demonstrates that Anekomochi suppresses postprandial glycemic response by enhancing insulin action rather than insulin secretion, through the promotion of GLP-1 release and activation of vagal afferent nerves. GLP-1 is well known as an incretin hormone that promotes insulin secretion via direct action on GLP-1 receptors expressed in pancreatic β cells [21, 22]. Although endogenous GLP-1 secreted from the intestines is extremely unstable, it acts on GLP-1 receptors expressed on vagal afferent nerves distributed near the intestines and hepatic portal vein, thereby promoting insulin secretion through neural pathways [33, 38–40]. Additionally, our recent studies have reported that the promotion of GLP-1 secretion by rare sugar D-allulose and gastrointestinal distension stimulates vagal afferent nerves, thereby enhancing insulin action [24, 26]. Enhancing insulin action is crucial for improving glucose intolerance, including the amelioration of insulin resistance. Therefore, the low postprandial glycemic response of Anekomochi, based on this mechanism, may be effective not only for healthy individuals but also for those with impaired glucose tolerance who have developed insulin resistance. Further research is needed to explore this potential.

One limitation of this study is that the rice samples were administered to mice without being heated with water. It was not feasible to administer a fixed amount of heated rice samples to the mice because the heated rice exhibited high viscosity. The primary carbohydrate in recommended laboratory diets for mice and rats, such as AIN-93 [41], is cornstarch (raw starch). Therefore, we determined that a single intragastric administration of an aqueous solution of raw rice samples represents a physiological condition for mice. However, in human diets, rice is typically consumed after being heated and cooked with water. Therefore, future research should investigate the effects of rice cooked with water on humans.

Rice is a staple grain in Asia, including Japan, and both non-glutinous (*uruchi*) and glutinous (*mochi*) rice are indispensable ingredients in Washoku, which was inscribed as a UNESCO Intangible Cultural Heritage in 2013. In recent years, rice, especially *mochi* rice, has been evaluated as a food with a high GI [16], leading health-conscious individuals and those with diabetes to avoid it. On the other hand, there are many cultivars of glutinous rice, and a review of various foods' GI values indicated that glutinous rice is not necessarily a high-GI food [17]. In this study, we demonstrated that some cultivars of glutinous rice, such as Anekomochi, exhibit low GI values. Habutaemochi, which is used in high-quality Japanese sweets due to its finer and smoother texture compared to other glutinous rice, exhibited a lower blood glucose response. The findings on the function of glutinous rice may contribute to preserving the culinary culture of Washoku, enhancing the enjoyment of rice-based meals, and improving the quality of life by encouraging the widespread use of glutinous rice as a food ingredient. Recent studies have reported that consumption of fish, meat, or milk before carbohydrates suppresses postprandial blood glucose elevation, an effect contributed to the enhancement of GLP-1 secretion by dietary protein and fat in these foods [42, 43]. Additionally, some glutinous rice cultivars also potently induce GLP-1 release. In the future, it may be possible to propose optimal dietary methods to prevent postprandial blood glucose elevation by analyzing the relationship between the timing of intake of foods with high GLP-1 secretion capacity, including glutinous rice, and their glycemic responses.

## Conclusions

Anekomochi exhibited the lowest postprandial glycemic response among the seven glutinous rice cultivars examined. In contrast, all three non-glutinous rice cultivars consistently exhibited a high glycemic response. The investigation into Anekomochi's effects revealed that it enhanced insulin action rather than promoting insulin secretion. This enhancement involved the release of intestinal GLP-1 and activation of the common hepatic branch of vagal afferent nerves, a target organ of endogenous intestinal GLP-1. These findings suggest that incorporating mochi rice into the diet could help maintain health and provide new strategies for the prevention and treatment of diabetes.

## Supplementary Information

Supplementary Figure 1.

Supplementary Figure 2.

## Data Availability

All data generated or analyzed during this study are included in this published article and its supplementary information files.

## Abbreviations

AUC: Area under the curve
BG: Blood glucose
Ex(9-39): Exendin(9-39)
GI: Glycemic index
GLP-1: Glucagon-like peptide-1
IP: Intraperitoneal
PO: Peroral
HOMA-IR: Homeostatic model assessment of insulin resistance
