# Supplementary material for: Anekomochi glutinous rice provides low postprandial glycemic response by enhanced insulin action via GLP-1 release and vagal afferents activation
Source: J Physiol Sci. 2024 Sep 27;74:47. doi: 10.1186/s12576-024-00940-5 (PMC11428336; doi:10.1186/s12576-024-00940-5)
Supplement: Supplementary file 1 — Supplementary Figure 1. [file 12576_2024_940_MOESM1_ESM.docx]

**Supplementary Figure 1. Individual data of changes in blood glucose levels after po administration of rice solutions shown in Figures 1 and 2.**

Hinohikari (2.59 g/kg, **A**), Kirara397 (2.79 g/kg, **B**), Nanatsuboshi (2.66 g/kg, **C**), Shimizumochi (2.61 g/kg, **D**), Habutaemochi (2.78 g/kg, **E**), Khao ha noi (2.50 g/kg, **F**), Anekomochi (2.71 g/kg, **G**), Akamochi (2.90 g/kg, **H**), Nioimochi (2.87 g/kg, **I**) or Hong Xie Nuo (2.65 g/kg, **J**) was po administered at 0 min in C57BL/6J mice fasted overnight. Sequential blood glucose levels (XY graphs) and their AUCs for the increase in blood glucose (AUC for ΔBG) during 0–120 min (bar graphs) were measured/calculated. The starch content was standardized to 2 g/kg in all groups. The numbers inside the bars indicate the sample size. n = 11–24. ***p* < 0.01*, *p* < 0.05 by two-way ANOVA followed by Bonferroni’s test (XY graphs) and ***p* < 0.01*, *p* < 0.05 by unpaired *t*-test (bar graphs).
