# Supplementary material for: Anekomochi glutinous rice provides low postprandial glycemic response by enhanced insulin action via GLP-1 release and vagal afferents activation
Source: J Physiol Sci. 2024 Sep 27;74:47. doi: 10.1186/s12576-024-00940-5 (PMC11428336; doi:10.1186/s12576-024-00940-5)
Supplement: Supplementary file 2 — Supplementary Figure 2. [file 12576_2024_940_MOESM2_ESM.docx]

**Supplementary Figure 2. The low postprandial glycemic response of Habutaemochi is also due to GLP-1 receptor signaling.**

Hinohikari (2.59 g/kg) or Habutaemochi (2.78 g/kg) was po administered at 0 min in C57BL/6J mice (**A**, **B**) or *Glp1r* KO mice (**C**, **D**), which were fasted overnight. n = 11–12. The data in **A**, **B** are re-analyses of the data presented in **Supplementary Figure 1A, E**. ***p* < 0.01 by two-way ANOVA followed by Bonferroni’s test in **A**. *P* value by unpaired *t*-test is shown in **B**.
